# *Moraxella* peritoneal dialysis-related infections: a systematic review

**DOI:** 10.1590/2175-8239-JBN-2025-0246en

**Published:** 2026-03-13

**Authors:** John Dotis, Charalampos Antachopoulos, Vasiliki Karava, Athina Papadopoulou, Nikoleta Printza

**Affiliations:** 1 Aristotle University of Thessaloniki, Hippokration Hospital, Third Department of Pediatrics, Thessaloniki, Greece.; 2 Aristotle University of Thessaloniki, Hippokration Hospital, First Department of Pediatrics, Thessaloniki, Greece.; 3 National and Kapodistrian University of Athens, Agia Sofia Children’s Hospital, First Department of Pediatrics, Athens, Greece.

**Keywords:** Moraxella, Peritonitis, Peritoneal Dialysis, Anti-Infective Agents, Susceptibility, Review

## Abstract

**Introduction:**

*Moraxella* species, although infrequent in peritoneal dialysis (PD)-associated infections, are established pathogens. *Moraxella catarrhalis* is recognized as a cause of upper and lower respiratory tract infections and is increasingly associated with β-lactamase production. The role of *Moraxella* species in PD-related infections warrants further investigation.

**Methods:**

A comprehensive search of PubMed and Google Scholar was conducted for cases of PD-associated infections caused by *Moraxella*. Inclusion criteria required a diagnosis of peritonitis or exit-site infection confirmed by culture. Demographic data, clinical presentation, microbiological identification, antimicrobial resistance, therapeutic regimens, and outcomes were analyzed.

**Results:**

Fourteen cases of *Moraxella*-related peritonitis in PD patients were identified between 1987 and 2024 from 13 studies, with no reports of exitsite infections. The mean age was 56 years; diabetic nephropathy was the most common underlying condition (four cases). *M. catarrhalis* (n = 6) and *Moraxella osloensis* (n = 5) were the most frequent pathogens. The most commonly reported methods were bacterial growth in culture media, followed by species identification using biochemical profiling or automated systems (n = 10), and advanced confirmatory techniques. Clinical presentation included abdominal pain, fever, and clouding of the dialysis fluid. Most isolates were susceptible to cephalosporins, aminoglycosides, and fluoroquinolones. The majority of patients (86%) retained their catheters after appropriate treatment, usually with intraperitoneal cephalosporins, achieving complete symptom resolution within a few days. Overall prognosis was favorable, with no mortality.

**Conclusion:**

*Moraxella* species, although rare, should be considered in the differential diagnosis of PD-associated infections. Identification using advanced techniques allows effective treatment and favorable outcomes, often without the need for catheter removal.

## Introduction

Peritoneal dialysis (PD) provides important benefits for patients with end-stage renal disease (ESRD), but its long-term efficacy is frequently compromised by infections, with peritonitis and exit-site infections being the most critical determinants of technique failure. While gram-positive organisms are the predominant causative pathogens, there is a growing recognition of infections caused by less common bacteria, including *Moraxella* species^1,2,3^.

*Moraxella* species are aerobic, gram-negative, oxidase-positive diplococci. The organism originally known as *Branhamella catarrhalis* was formally reclassified under the genus *Moraxella* in 1984, thereby receiving its current name, *Moraxella catarrhalis* ^4^. This taxonomic change was later supported by DNA-hybridization studies and 16S rRNA gene sequencing, which demonstrated a closer genetic relationship to the genus *Moraxella* ^2,5^. Although typically commensals of the human respiratory tract and mucosal surfaces, these bacteria have increasingly been recognized as rare yet significant pathogens in cases of peritonitis among patients undergoing peritoneal dialysis. Furthermore, *M. catarrhalis* has been identified as a causative agent of PD-associated peritonitis^6^. Additionally, other species such as *Moraxella osloensis* have also been implicated, albeit less frequently^1^. The emergence of these species as causative pathogens of PD-associated peritonitis is noteworthy, given their rarity and the diagnostic challenges they present.

The pathogenesis of *Moraxella*-induced peritonitis in PD patients is not fully elucidated. Proposed mechanisms include direct contamination during catheter insertion or manipulation, hematogenous spread from distant sites, or translocation from the gastrointestinal tract^3,6,7^. Accurate identification of *Moraxella* species requires initial growth on conventional culture media, followed by advanced methods for species-level confirmation. Techniques such as matrix-assisted laser desorption/ionization time-of-flight mass spectrometry (MALDI-TOF MS) and 16S ribosomal RNA (16S rRNA) gene sequencing are ideal for correct species identification, especially when phenotypic similarities with other gramnegative organisms may lead to misclassification^2^.

*Moraxella* species are generally resistant to penicillins due to the high prevalence of β-lactamase production; therefore, penicillins cannot be considered reliable treatment options. Resistance patterns may vary, and empirical therapy should be guided by antimicrobial susceptibility testing, local antibiograms, and individual patient factors^5^. The management approach often includes intraperitoneal antibiotic administration, with the duration tailored to clinical response^8^.

This review aims to synthesize and critically evaluate the existing literature on *Moraxella*-associated infections in PD patients, with a focus on clinical manifestations, diagnostic challenges, microbiological assessment, treatment strategies and outcomes. By analyzing reported cases, we seek to deepen the understanding of these uncommon infections and inform clinical practice to improve patient care.

## Methods

### Study Framework and Protocol Registration

This systematic review was structured in accordance with the PRISMA 2020 guidelines for transparent reporting of systematic evidence syntheses^9^. The review protocol was prospectively registered in the PROSPERO international prospective registry of systematic reviews (ID: CRD420251042629; accessible at: https://www.crd.york.ac.uk/PROSPERO/view/CRD420251042629, accessed April 29, 2025).

### Selection Parameters

We included published case reports documenting infectious complications in patients undergoing peritoneal dialysis (PD), specifically peritonitis and/or exit-site infections (ESIs), attributed to *Moraxella* species. The diagnosis of peritonitis was established when at least two of the following criteria were met: (i) clinical features consistent with peritonitis, such as abdominal pain and/or cloudy dialysis effluent; (ii) peritoneal fluid white cell count >100/µL with >50% polymorphonuclear leukocytes after a dwell time of at least 2 hours; (iii) positive dialysis effluent culture^8^. Exit-site infections were defined as the presence of purulent exudate at the catheter exit-site, irrespective of the presence or absence of pericatheter erythema. Tunnel infections, often clinically silent, were considered when inflammation was observed along the catheter’s subcutaneous course, particularly in conjunction with ESI^8^. Only culture-confirmed infections due to *Moraxella* species from dialysate, exit-site exudate, or tunnel tissue were included. Pathogen identification was required to be based on conventional culture-based biochemical methods, while additional confirmatory techniques such as MALDI-TOF MS and/or 16S rRNA gene sequencing were accepted when reported.

### Data Acquisition Strategy

An extensive search of the medical literature was conducted using two electronic databases: PubMed and Google Scholar. The search covered all literature published up to May 1, 2025. Keywords included: “*Moraxella*” or “*Branhamella*” associated with “peritonitis”, “exit-site infection”, and “peritoneal dialysis”. No language restrictions were applied. Reports in languages other than English were translated using Google Translate (https://translate. google.gr, last accessed May 31, 2025).

### Screening and Eligibility Assessment

Two stages of screening were applied. Initially, titles and abstracts were reviewed independently by two investigators (JD and AP) for potential relevance. The full texts of eligible articles were then assessed independently by three additional reviewers (CA, VK, and NP). Any discrepancies were resolved via discussion among all reviewers (JD, CA, VK, AP, NP) to ensure consensus. Reference lists of included studies were manually reviewed to identify additional cases.

### Data Extraction and Management

All relevant case data were extracted into a structured database. No reports were excluded based on completeness, although some lacked specific clinical or microbiological details. Extracted variables included publication year, geographic origin, patient demographics (age, sex), primary renal diagnosis leading to ESRD, prior episodes of PD-related infections, clinical presentation, laboratory and microbiological findings, diagnostic modalities, antimicrobial susceptibility profiles, treatment regimens (drug type, route, duration), catheter management strategies, and clinical outcomes.

### Ethical Compliance

This article is based on previously published data and does not contain any new studies with human participants or animals performed by any of the authors. Therefore, ethical approval and informed consent were not applicable.

### Data Synthesis and Statistical Analysis

A centralized dataset was constructed using Microsoft Excel (version 5.2.3790.1830, Microsoft Corporation, Redmond, WA, USA). Quantitative variables were summarized descriptively. Statistical analysis was performed using GraphPad Instat (version 3.10; GraphPad Software, San Diego, CA, USA).

## Results

**Figure 1:**
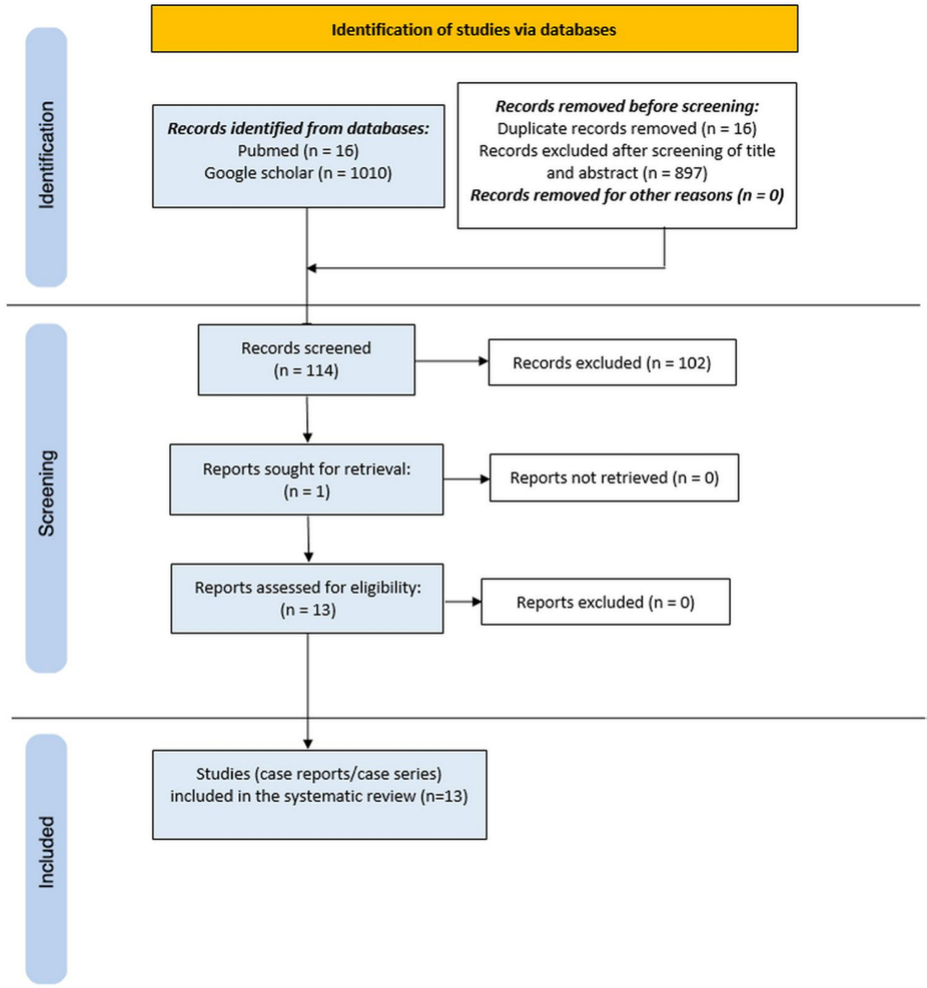
The PRISMA flow diagram of our literature search and selection process applied during the overview.

Our systematic search yielded 1,026 records, of which 114 remained following removal of duplicates. After title and abstract review, 13 case reports/series were included for full-text assessment, as depicted in the PRISMA flow diagram (Figure 1). Ultimately, a total of 14 distinct cases of PD-associated infections caused by *Moraxella* species were identified, all of which were cases of peritonitis. All infections originated in the outpatient setting and none were associated with hospital care. These cases spanned the period from 1987 to 2024 and were reported across seven different countries in North America, Europe, and Asia, with the majority originating from the United States.

The included cases involved 9 male and 5 female patients with a wide age distribution (range: 22–83 years; median: 56 years). All patients were undergoing PD for a dialysis vintage period ranging from 12 to 84 months (Table 1). Diabetic nephropathy was the most frequently reported primary renal pathology (n = 4), followed by single cases of hypertensive renal disease, membranous nephropathy, malignant nephrosclerosis, and chronic glomerulonephritis in patients with available data. Notably, none of the patients were reported to have significant comorbidities, immunosuppression, or other systemic health issues apart from the underlying nephrological condition necessitating PD. This may suggest that *Moraxella*-associated peritonitis can also occur in immunocompetent individuals. A history of prior peritonitis episodes was noted in the majority (56%; 5/9) of cases, typically involving common pathogens such as *Staphylococcus aureus* or *Streptococcus* species.

**Table 1:** Reported cases of peritoneal dialysis-associated infections caused by *Moraxella* species, listed chronologically from oldest to most recent

No.	Publication year, language, reference	Country	Age (y)	Gender	Type of infection	Vintage on PD (months), (etiology)	PD method	Previous peritonitis
1	1987, English^10^	UK	51	F	Peritonitis	60 (diabetic nephropathy)	CAPD	NA
2	1990, English^11^	USA	22	F	Peritonitis	NA	CAPD	2 peritonitis episodes (*Staphylococcus aureus*)
3	1990, English^11^	USA	65	F	Peritonitis	NA	CAPD	1 peritonitis episode (*Staphylococcus aureus*)
4	1993, English^12^	USA	46	M	Peritonitis	48 (diabetic nephropathy)	CAPD	2 peritonitis episodes (1^st^ *Staphylococcus haemolyticus*, 2^nd^ a-haemolytic *streptococcus*)
5	2003, English^13^	USA	48	M	Peritonitis	NA	CAPD	NA
6	2009, English^14^	Italy	70	F	Peritonitis	60 (hypertensive renal disease)	CCPD	3 bacterial peritonitis episodes
7	2012, English^6^	USA	56	M	Peritonitis	NA	PD	No
8	2014, English^1^	Netherlands	47	M	Peritonitis	53 (membranous nephropathy)	CAPD	1 peritonitis episode (*Streptococcus gordonii,* 36 months previous)
9	2014, English^15^	Spain	73	M	Peritonitis	24 (diabetic nephropathy)	PD	NA
10	2016, Spanish^16^	Spain	83	M	Peritonitis	NA	PD	NA
11	2018, English^17^	USA	68	M	Peritonitis	24 (NA)	APD	No
12	2018, English^18^	Serbia	59	M	Peritonitis	12 (diabetic nephropathy)	CAPD	No
13	2019, English^2^	Japan	26	M	Peritonitis	14 (malignant nephrosclerosis)	CAPD	No
14	2024, English^3^	Japan	46	F	Peritonitis	84 (chronic glomerulonephritis)	PD	NA

Abbreviations – PD: peritoneal dialysis; F: female; M: male; NA: not available; CAPD: continuous ambulatory peritoneal dialysis; CCPD: continuous cycling peritoneal dialysis; APD: ambulatory peritoneal dialysis.

Among the 14 patients with *Moraxella*-associated peritonitis, information on the presence of fever was available for 12. Of these, 7 (58%) were afebrile, while 5 (42%) exhibited febrile responses, with temperatures ranging from 37.8 to 38.6°C or simply noted as “fever”. Peritoneal white blood cell (WBC) counts showed considerable variability, ranging from 260 to 10,528 cells/mm^3^. A predominance of polymorphonuclear cells (PMNs) was observed in 8 cases (>90%), with the highest recorded proportion reaching 99%. One case had a notably low PMN percentage (15%), and differential counts were unavailable in two cases. Peripheral blood WBC counts were available only in 6 cases and ranged from 1,675 to 10,500 × 10^6^/L. In most of these, a neutrophilic predominance was noted, with PMN percentages ranging from 78 to 92%, except for one case with a notably low neutrophil proportion (37%).

*M. catarrhalis* was the predominant isolate (n = 6), followed by *M. osloensis* (n = 5), while *Moraxella phenylpyruvica* and *Moraxella nonliquefaciens* were each identified in a single case (Table 2). An additional case involved an unidentified *Moraxella* species. Conventional culture methods supported bacterial growth (e.g., BacT/Alert systems, chocolate agar, blood agar), followed by species identification through biochemical profiling or automated identification systems (n = 10). In more recent reports, advanced confirmatory techniques such as MALDI-TOF MS (n = 4) and/or 16S rRNA gene sequencing (n = 3) were additionally employed, reflecting the evolution of microbiological diagnostic capabilities over time. Five cases reported polymicrobial infections, including co-isolation of *Enhydrobacter aerosaccus*, *Kluyvera ascorbata*, *Rhizobium radiobacter*, mixed *Streptococci/ Enterococci* and diphtheroids. These cases emphasized the need for comprehensive microbiological workup, particularly in patients with atypical clinical courses.

**Table 2:** Analysis of microbiological data from 14 peritoneal dialysis-related peritonitis cases attributed to *Moraxella* species

No (ref)	*Moraxella* isolate	Onset to treatment (d)	Isolation method	Co-infection	Antimicrobial sensitivity pattern	Antimicrobial resistant pattern
1^10^	*Branhamella catarrhalis*	1	Culture of PD effluent	No	penicillin, cefuroxime, gentamicin, erythromycin, chloramphenicol	NA
2^11^	*Branhamella catarrhalis*	3	Culture of PD effluent	No	ampicillin, cephalothin, tetracycline, amikacin, gentamicin, tobramycin, mezlocillin, piperacillin, chloramphenicol	trimethoprim/sulfamethoxazole
3^11^	*Branhamella catarrhalis*	1	Culture of PD effluent	No	cefotaxime, gentamicin, chloramphenicol, trimethoprim/sulfamethoxazole	ampicillin, penicillin, vancomycin
4^12^	*Moraxella catarrhalis*	2	Culture of PD effluent (BacT/Alert, Chocolate agar, blood agar)	No	amoxicillin/clavulanic acid, ampicillin/sulbactam, cefotaxime, ceftriaxone, cefuroxime, imipenem, erytromycin, chloramphenicol, gentamicin, trimethoprim/sulfamethoxazole	ampicillin
5^13^	*Moraxella* unidentified specie	NA	Culture of PD effluent	*Kluyvera ascorbata*	NA	NA
6^14^	*Moraxella phenylpyruvica*	NA	Culture of PD effluent, Versa TREK, BBL Crystal ID	No	NA	NA
7^6^	*Moraxella catarrhalis*	14	Culture of PD effluent (Chocolate agar, blood agar)	Diptheroids	amoxicillin/clavulanic acid, ceftazidime, ceftriaxone, cefuroxime, clarithromycin, ciprofloxacin, levofloxacin, tetracycline, trimethoprim/sulfamethoxazole	NA
8^1^	*Moraxella osloensis*	7	MALDI-TOF MS	*Rhizobium radiobacter*	All antimicrobials	No
9^15^	*Moraxella nonliquefaciens*	NA	Culture of PD effluent (BacT/Alert, Chocolate agar, blood agar), Vitek-2, 16S rRNA gene sequencing	No	amoxicillin/clavulanic acid, cotrimoxazole, azithromycin	NA
10^16^	*Moraxella osloensis*	2	MALDI-TOF MS	Streptococci/Enterococci	amoxicillin, amoxicillin/clavulanic acid, cefuroxime, cefotaxime, ceftazidime, ciprofloxacin, gentamicin, azithromycin	vancomycin, teicoplanin, daptomycin
11^17^	*Moraxella osloensis*	1	16S rRNA gene sequencing	No	NA	NA
12^18^	*Moraxella catarrhalis*	1	Culture of PD effluent (BacT/Alert)	No	ampicillin, amoxicillin/clavulanic acid, cefazolin, ceftazidime, ceftriaxone, erythromicin, trimethoprim/sulfamethoxazole, tetracycline	vancomycin
13^2^	*Moraxella osloensis*	2	MALDI-TOF MS, 16S rRNA gene sequencing	*Enhydrobacter aerosaccus*	ampicillin, cefaclor, cefotiam, ceftriaxone, cefepime, imipenem, clarithromycin, levofloxacin, minocycline	clindamycin
14^3^	*Moraxella osloensis*	10	MALDI-TOF MS	No	ampicillin/sulbactam, cefotaxime, ceftazidime, ceftriaxone, cefepime, imipenem, meropenem, erythromycin, clarithromycin, levofloxacin	penicillin G, ampicillin, clindamycin, vancomycin, linezolid, daptomycin

Abbreviations – PD: peritoneal dialysis; NA: not available; BacT/Alert and Versa TREK: automated microbial detection systems; BBL Crystal ID and Vitek-2: microbial identification systems; MALDI-TOF MS: matrix-assisted laser-desorption ionization time – flight mass spectrometry; 16S rRNA gene sequencing: 16S ribosomal RNA gene sequencing.

Antibiotic sensitivity data were available for 11 isolates. Most isolates were susceptible to third-generation cephalosporins (e.g., cefotaxime, ceftriaxone, ceftazidime), aminoglycosides (e.g., gentamicin, tobramycin, amikacin), macrolides (e.g., erythromycin, clarithromycin), and fluoroquinolones (e.g., ciprofloxacin, levofloxacin). However, resistance to penicillin and ampicillin was noted in a few cases, particularly among *M. catarrhalis* and *M. osloensis* strains, consistent with their known production of β-lactamase enzymes.

In polymicrobial infections, the choice of antibiotic therapy was guided by the most resistant co-isolated organism. Notably, in one case involving co-isolation with *Rhizobium radiobacter*, resistance to multiple β-lactams necessitated the use of intraperitoneal ciprofloxacin and intravenous meropenem.

All patients with complete reported data received intraperitoneal antibiotics as part of their initial treatment protocol. The most commonly used agents (e.g., cefazolin, ceftazidime) were often in combination with aminoglycosides or fluoroquinolones (Table 3). Treatment durations ranged from 10 to 28 days, with a median of 21 days. Catheter removal was required in only two cases, one involving *Moraxella phenylpyruvica* and the other *M. osloensis,* both of which resulted in a transition to hemodialysis. In all 14 cases, the infection was resolved without mortality or long-term complications. Notably, catheter salvage was achieved in 86% (12/14) of patients, reaffirming the generally favorable prognosis of *Moraxella*-related PD infections when promptly recognized and adequately treated.

**Table 3:** Therapeutic strategies and clinical outcomes in reviewed cases of *Moraxella*-associated peritonitis in patients undergoing peritoneal dialysis

No.^(ref)^	Catheter removal	Hemodialysis switch	Treatment (duration)	Outcome
1^10^	No	No	ip cefuroxime + ip gentamicin (10 days)	Recovered
2^11^	No	No	ip vancomycin + iv vancomycin ≥ im penicillin G + ip penicillin G	Recovered
3^11^	No	No	ip vancomycin + iv vancomycin ≥ iv gentamicin + ip gentamicin + ip trimethoprim/sulfamethoxazole + iv trimethoprim/sulfamethoxazole + iv ceftriaxone	Recovered
4^12^	No	No	im cefazolin + im tobramycin ≥ ip cefazolin (10 days) + ip tobramycin	Recovered
5^13^	No	No	levofloxacin + ip tobramycin (14 days)	Recovered
6^14^	Yes (day 5)	Yes	ip tobramycin ≥ vancomycin + fluconazole ≥ pos cefixime	Recovered
7^6^	No	No	ip vancomycin + iv ciprofloxacin ≥ ip vancomycin + ip ceftazidime (21 days)	Recovered
8^1^	Yes (day 12)	Yes	ip cefazolin ≥ ip ceftazidime ≥ ip ciprofloxacin ≥ iv meropenem	Recovered
9^15^	No	No	ip cefazolin + ip ceftazidime ≥ ip ceftazidime (28 days)	Recovered
10^16^	No	No	ip cefazolin + ip ceftazidime + pos linezolid ≥ ip ceftazidime + pos amoxicillin/clavulanic acid	Recovered
11^17^	No	No	ip ceftazidime (21 days)	Recovered
12^18^	No	No	ip cefazolin + ip amikacin ≥ ip cefazolin (14 days)	Recovered
13^2^	No	No	ip ceftazidime + ip cefazolin ≥ ip cefazolin (21 days)	Recovered
14^3^	No	No	ip ceftazidime + ip cefazolin ≥ ip ceftazidime (21 days)	Recovered

Abbreviations – ip: intraperitoneal; iv: intravenous; im: intramuscular; pos: per os.

The overall prognosis was favorable across all reported cases. No infection-related mortality was documented. Clinical resolution of peritonitis symptoms was typically achieved within few days of antibiotic initiation. Laboratory parameters, including peritoneal fluid white blood cell counts and serum inflammatory markers, showed rapid improvement in most cases.

## Discussion

This systematic review comprehensively examines the role of *Moraxella* species as etiological agents in peritoneal dialysis-associated infections, with a specific focus on peritonitis. The exclusive documentation of *Moraxella*-related cases as peritonitis emphasizes their clinical infrequency, the inherent diagnostic complexities, and the generally favorable therapeutic outcomes reported. The findings underscore the importance of maintaining a high index of suspicion for atypical pathogens such as *Moraxella* species, which, despite their rarity, deserve clinical recognition in the differential diagnosis of PD-related peritonitis. Their identification has historically been overlooked, likely due to limitations of traditional diagnostic methodologies and their commensal nature in mucosal sites.

A consistent observation across cases was the predominance of *M. catarrhalis* and *M. osloensis*, both traditionally associated with respiratory tract colonization. Their isolation from peritoneal fluid raises intriguing questions regarding pathogenic mechanisms. Several routes of peritoneal cavity colonization have been proposed, including direct contamination during PD exchanges, translocation from the gastrointestinal tract, and hematogenous spread from distant mucosal foci^3,6,7^. The presence of co-infecting organisms in some cases may suggest a polymicrobial origin or reflect compromised mucosal barriers.

Importantly, the review highlights the evolving microbiological armamentarium in detecting *Moraxella* species. While earlier cases relied solely on biochemical testing and growth on selective media, more recent cases incorporated MALDI-TOF MS and 16S rRNA gene sequencing for accurate specieslevel identification^19^. These advancements have markedly enhanced the sensitivity and specificity of pathogen detection and may partly explain the increased detection rate of *Moraxella* species in more recent years^2^. Nevertheless, despite technological progress, diagnostic delays remain possible in low-resource settings. From a clinical standpoint, heightened awareness is necessary for early recognition of *Moraxella*-associated PD peritonitis. Infections due to fastidious organisms may present with subacute symptoms or atypical laboratory profiles, leading to misclassification or underdiagnosis.

The antimicrobial susceptibility profile of *Moraxella* isolates in PD-associated infections was generally favorable. Most strains exhibited susceptibility to cephalosporins, aminoglycosides and fluoroquinolones, consistent with the known antibiotic profiles of *Moraxella* species. In particular, *M. catarrhalis*, despite its ability to produce β-lactamase enzymes, demonstrated preserved susceptibility to cephalosporins. Nonetheless, resistance to penicillin and ampicillin was reported in few isolates of both *M. catarrhalis* and *M. osloensis,* emphasizing the importance of individualized antimicrobial therapy guided by susceptibility testing^2,5^.

Despite the presence of isolates with variable susceptibility to antibiotics, clinical outcomes were uniformly favorable, with all 14 patients achieving resolution of infection. Most infections were successfully managed without catheter removal, a noteworthy outcome considering that catheter salvage is a critical determinant of long-term PD continuation. Only two patients (14%) required catheter removal, both of whom eventually resumed peritoneal dialysis following switch to hemodialysis. This reaffirms prior observations that even non-traditional pathogens can be effectively managed with early intervention and appropriate therapy^20^.

Therapeutic strategies varied in drug choice and duration, reflecting empirical decisions tailored to patient condition and local practices. Notably, intraperitoneal cephalosporins, often in combination with aminoglycosides or fluoroquinolones, were the most commonly employed regimens. Treatment duration was typically aligned with standard ISPD guidelines for gram-negative peritonitis, ranging from 14 to 21 days^8^. The consistent recovery in our cohort suggests that *Moraxella* infections in PD patients follow a benign course when managed according to protocol.

An additional point of consideration is the potential impact of polymicrobial infections. In 5 of the 14 reviewed cases (~36%), *Moraxella* species were co-isolated with other microorganisms in peritoneal fluid cultures, complicating the interpretation of microbiological findings and the determination of the primary causative agent. The co-isolated organisms including *Enhydrobacter aerosaccus*, *Kluyvera ascorbate*, and *Rhizobium radiobacter* are rarely reported or poorly characterized as etiological agents in PD-related peritonitis. Among them, *R. radiobacter* has occasionally been implicated in PD peritonitis, primarily in immunocompromised hosts or in the context of catheter-related infections^21^. In contrast, *K. ascorbata* and *E. aerosaccus* are considered organisms of low virulence, with only anecdotal or exceptional associations with human disease and virtually no established link to PD-associated infections. Mixed *Streptococci/Enterococci* and diphtheroids, although more frequently encountered in clinical specimens, are often regarded as contaminants, albeit with potential pathogenicity in selected cases^8^. As a result, the pathogenic role of *Moraxella* in these polymicrobial episodes remains unclear. Whether it acts as a true pathogen or merely as a commensal cannot be determined without more detailed clinical, inflammatory, and microbiological data. Nevertheless, these mixed infections may obscure diagnosis and delay targeted therapy. Importantly, in all polymicrobial cases, culture-guided antimicrobial treatment led to clinical resolution, underscoring the critical importance of thorough microbiological evaluation, particularly in cases with atypical presentations or suboptimal initial response to empirical therapy^22^.

Our study has certain unavoidable limitations, primarily due to its reliance on previously published case reports rather than original patient data. As a result, it is susceptible to publication bias and may not represent the complete range of clinical scenarios. In addition, heterogeneity in reporting, particularly with regard to susceptibility testing and treatment regimens, may limit generalizability. Nonetheless, this review provides the most comprehensive analysis to date on *Moraxella* peritonitis in PD and offers clinically relevant guidance.

In conclusion, although rare, *Moraxella* species are recognized pathogens in the setting of PD-associated peritonitis. Diagnosis should be based on clinical features, peritoneal fluid analysis, and culture results. Prompt identification using conventional culture methods, with advanced confirmatory techniques when available, along with appropriate antimicrobial therapy and judicious catheter management, can result in uniformly favorable clinical outcomes. As diagnostic tools become more sophisticated, clinicians should consider them and integrate them into laboratory practice. Further research is warranted to define the true incidence of *Moraxella* peritoneal dialysis-related infections, optimal management strategies, and the long-term impact on peritoneal dialysis outcomes.

## Data Availability

No new data were generated or analyzed in this study.
