# Supplementary material for: Moraxella peritoneal dialysis-related infections: a systematic review
Source: J Bras Nefrol. 2026 Mar 13;48(2):e20250246. doi: 10.1590/2175-8239-JBN-2025-0246en (PMC12987636; doi:10.1590/2175-8239-JBN-2025-0246en)
Supplement: Translation

# Translation: Infecções por *Moraxella* relacionadas à diálise peritoneal: uma revisão sistemática

**DOI:** 10.1590/2175-8239-JBN-2025-0246pt

**Authors:** John Dotis, Charalampos Antachopoulos, Vasiliki Karava, Athina Papadopoulou, Nikoleta Printza

**Affiliations:** 1 Aristotle University of Thessaloniki, Hippokration Hospital, Third Department of Pediatrics, Thessaloniki, Grécia.; 2 Aristotle University of Thessaloniki, Hippokration Hospital, First Department of Pediatrics, Thessaloniki, Grécia.; 3 National and Kapodistrian University of Athens, Agia Sofia Children’s Hospital, First Department of Pediatrics, Athens, Grécia.

**Keywords:** Moraxella, Peritonite, Diálise Peritoneal, Suscetibilidade, Anti-Infecciosos, Revisão

## Abstract

**Introdução:**

Espécies de *Moraxella*, embora raras em infecções associadas à diálise peritoneal (DP), são patógenos estabelecidos. *Moraxella catarrhalis* causa infecções respiratórias, frequentemente associadas à produção de β-lactamase. Seu papel na DP merece investigação.

**Métodos:**

Buscou-se nas bases PubMed e Google Scholar casos de infecções por *Moraxella* em DP. Critérios de inclusão: diagnóstico de peritonite ou infecção no local de saída confirmados por cultura. Analisaramse dados demográficos, clínicos, microbiologia, resistência, terapêutica e desfechos.

**Resultados:**

Catorze casos de peritonite relacionada à *Moraxella* em pacientes em DP foram identificados entre 1987 e 2024, a partir de 13 estudos, sem relatos de infecções no local de saída. A idade média foi 56 anos; a nefropatia diabética foi a condição de base mais comum (quatro casos). *M. catarrhalis* (n = 6) e *Moraxella osloensis* (n = 5) foram os patógenos mais frequentes, com crescimento bacteriano apoiado em meios de cultura, seguido de identificação por perfis bioquímicos ou sistemas automatizados (n = 10), e técnicas confirmatórias avançadas empregadas quando relatadas. A apresentação clínica incluiu dor abdominal, febre e turbidez do líquido de diálise. A maioria dos isolados mostrou suscetibilidade a cefalosporinas, aminoglicosídeos e fluoroquinolonas. A maior parte dos pacientes (86%) manteve os cateteres após tratamento adequado, geralmente com administração intraperitoneal de cefalosporinas, com resolução completa dos sintomas em poucos dias. O prognóstico geral foi favorável, sem mortalidade.

**Conclusões:**

Espécies de *Moraxella*, embora raras, devem constar no diagnóstico diferencial das infecções relacionadas à DP. Sua identificação por técnicas avançadas permite tratamento eficaz e desfechos favoráveis, frequentemente sem necessidade de remoção do cateter.

## Introdução

A diálise peritoneal (DP) oferece benefícios importantes para pacientes com doença renal em estágio terminal (DRET); no entanto, sua eficácia a longo prazo é frequentemente comprometida por infecções, sendo a peritonite e as infecções no sítio de saída os determinantes mais críticos de falha da técnica. Embora os organismos gram-positivos sejam os patógenos causadores predominantes, há um reconhecimento crescente das infecções causadas por bactérias menos comuns, incluindo as espécies de *Moraxella* ^1,2,3^.

As espécies de *Moraxella* são diplococos aeróbicos, gram-negativos e oxidase-positivos. O organismo originalmente conhecido como *Branhamella catarrhalis* foi formalmente reclassificado no gênero *Moraxella* em 1984, passando a receber a denominação atual de *Moraxella catarrhalis* ^4^. Essa alteração taxonômica foi posteriormente corroborada por estudos de hibridização de DNA e sequenciamento do gene 16S rRNA, que demonstraram uma relação genética mais estreita com o gênero *Moraxella* ^2,5^. Embora sejam tipicamente comensais do trato respiratório humano e das superfícies mucosas, essas bactérias têm sido cada vez mais reconhecidas como patógenos raros, porém significativos, em casos de peritonite entre pacientes em diálise peritoneal. Além disso, a *M. catarrhalis* foi identificada como agente etiológico da peritonite associada à DP^6^. Adicionalmente, outras espécies, como *Moraxella osloensis*, também têm sido implicadas, embora com menor frequência^1^. O surgimento dessas espécies como patógenos causadores de peritonite associada à DP é digno de nota, dada sua raridade e os desafios diagnósticos que apresentam.

A patogênese da peritonite induzida por *Moraxella* em pacientes em DP não está completamente elucidada. Os mecanismos propostos incluem contaminação direta durante a inserção ou manipulação do cateter, disseminação hematogênica a partir de sítios distantes ou translocação a partir do trato gastrointestinal^3,6,7^. A identificação precisa das espécies de *Moraxella* requer crescimento inicial em meios de cultura convencionais, seguido de métodos avançados para confirmação em nível de espécie. Técnicas como espectrometria de massa por ionização/dessorção a laser assistida por matriz com tempo de voo (MALDI-TOF MS) e sequenciamento do gene do RNA ribossômico 16S (16S rRNA) são ideais para a identificação correta de espécies, especialmente quando semelhanças fenotípicas com outros organismos gram-negativos podem levar a classificações incorretas^2^.

As espécies de *Moraxella* são geralmente resistentes às penicilinas devido à elevada prevalência da produção de β-lactamase; portanto, as penicilinas não podem ser consideradas opções terapêuticas seguras. Os padrões de resistência podem variar, e a terapia empírica deve ser orientada por testes de suscetibilidade antimicrobiana, antibiogramas locais e fatores individuais do paciente^5^. O manejo terapêutico geralmente inclui a administração intraperitoneal de antibióticos, sendo a duração ajustada à resposta clínica^8^.

Esta revisão tem como objetivo sintetizar e avaliar criticamente a literatura existente sobre infecções associadas à *Moraxella* em pacientes em DP, com foco nas manifestações clínicas, nos desafios diagnósticos, na avaliação microbiológica, nas estratégias de tratamento e nos desfechos. Ao analisar os casos relatados, buscamos aprofundar a compreensão dessas infecções incomuns e contribuir para a prática clínica, a fim de melhorar o cuidado ao paciente.

## Métodos

### Estrutura do Estudo e Registro do Protocolo

Esta revisão sistemática foi estruturada de acordo com as diretrizes PRISMA 2020 para o relato transparente de sínteses sistemáticas de evidências^9^. O protocolo da revisão foi registrado prospectivamente no PROSPERO (registro internacional prospectivo de revisões sistemáticas) (ID: CRD420251042629; disponível em: https://www.crd.york.ac.uk/PROSPERO/view/CRD420251042629, acedido em 29 de abril de 2025).

### Parâmetros de Seleção

Foram incluídos relatos de casos publicados, que documentaram complicações infecciosas em pacientes submetidos à diálise peritoneal (DP), especificamente peritonite e/ou infecções do sítio de saída (ESI, do inglês *exit-site infections*), atribuídas a espécies de *Moraxella*. O diagnóstico de peritonite foi estabelecido quando pelo menos dois dos seguintes critérios foram atendidos: (i) características clínicas consistentes com peritonite, como dor abdominal e/ou efluente de diálise turvo; (ii) contagem de leucócitos no líquido peritoneal >100/µL com >50% de leucócitos polimorfonucleares após um tempo de permanência de pelo menos 2 horas; (iii) cultura positiva do efluente de diálise^8^. As infecções do sítio de saída foram definidas como a presença de exsudato purulento no sítio de saída do cateter, independentemente da presença ou ausência de eritema pericateter. Infecções do túnel, muitas vezes clinicamente silenciosas, foram consideradas quando se observou inflamação ao longo do trajeto subcutâneo do cateter, particularmente em conjunto com a ESI^8^. Foram incluídas somente as infecções confirmadas por cultura atribuídas a espécies de *Moraxella*, provenientes do dialisato, do exsudato do sítio de saída ou do tecido do túnel. A identificação do patógeno deveria ter como base métodos bioquímicos convencionais dependentes de cultura, sendo aceitas técnicas confirmatórias adicionais, como MALDI-TOF MS e/ou sequenciamento do gene 16S rRNA, quando relatadas.

### Estratégia de Aquisição de Dados

Foi realizada uma busca abrangente na literatura médica utilizando duas bases de dados eletrônicas: PubMed e Google Scholar. A busca contemplou toda a literatura publicada até 1º de maio de 2025. As palavras-chave incluíram: “*Moraxella*” ou “*Branhamella*” associadas aos termos “peritonite”, “infecção do sítio de saída” e “diálise peritoneal”. Não foram aplicadas restrições de idioma. Os relatos publicados em outros idiomas além do inglês foram traduzidos por meio do *Google Translate* (https://translate.google.gr, acedido pela última vez em 31 de maio de 2025).

### Triagem e Avaliação de Elegibilidade

Foram aplicadas duas etapas de triagem. Inicialmente, os títulos e resumos foram revisados independentemente por dois pesquisadores (JD e AP) quanto à possível relevância. Em seguida, os textos completos dos artigos elegíveis foram avaliados de forma independente por três revisores adicionais (CA, VK e NP). Quaisquer discrepâncias foram resolvidas por meio de discussão entre todos os revisores (JD, CA, VK, AP e NP), de modo a garantir o consenso. As listas de referências dos estudos incluídos foram revisadas manualmente para identificar casos adicionais.

### Extração e Manejo de Dados

Todos os dados relevantes dos casos foram extraídos para um banco de dados estruturado. Nenhum relato foi excluído com base na completude das informações, embora alguns não apresentassem detalhes clínicos ou microbiológicos específicos. As variáveis extraídas incluíram ano de publicação, origem geográfica, dados demográficos dos pacientes (idade, sexo), diagnóstico renal primário que levou à DRET, episódios anteriores de infecções relacionadas à DP, apresentação clínica, achados laboratoriais e microbiológicos, modalidades diagnósticas, perfis de suscetibilidade antimicrobiana, esquemas terapêuticos (tipo de medicamento, via de administração, duração), estratégias de manejo do cateter e desfechos clínicos.

### Conformidade Ética

Este artigo baseia-se em dados previamente publicados e não inclui novos estudos envolvendo seres humanos ou animais realizados por qualquer um dos autores. Portanto, a aprovação por comitê de ética e a obtenção de consentimento informado não foram necessárias.

### Síntese de Dados e Análise Estatística

Um conjunto de dados centralizado foi construído utilizando o Microsoft Excel (versão 5.2.3790.1830, *Microsoft Corporation*, Redmond, WA, EUA). As variáveis quantitativas foram resumidas de forma descritiva. A análise estatística foi realizada com o *GraphPad Instat* (versão 3.10; *GraphPad Software*, San Diego, CA, EUA).

## Resultados

Nossa busca sistemática resultou em 1.026 registros, dos quais 114 permaneceram após a remoção de duplicatas. Após a revisão de títulos e resumos, 13 relatos/séries de casos foram incluídos para avaliação do texto na íntegra, conforme ilustrado no fluxograma PRISMA (Figura 1). Ao final, foram identificados 14 casos distintos de infecções associadas à DP causadas por espécies de *Moraxella*, todos correspondentes a episódios de peritonite. Todas as infecções tiveram origem em ambientes ambulatoriais e nenhuma foi associada a cuidados hospitalares. Esses casos abrangeram o período de 1987 a 2024 e foram relatados em sete países diferentes na América do Norte, Europa e Ásia, sendo a maioria proveniente dos Estados Unidos.

**Figura 1:** Fluxograma PRISMA da busca bibliográfica e do processo de seleção aplicado ao longo da revisão.

Os casos incluídos envolveram 9 pacientes do sexo masculino e 5 do sexo feminino, com uma ampla distribuição etária (variação: 22–83 anos; mediana: 56 anos). Todos os pacientes estavam em DP, com duração de tratamento variando de 12 a 84 meses (Tabela 1). A nefropatia diabética foi a patologia renal primária mais frequentemente relatada (n = 4), seguida por casos isolados de doença renal hipertensiva, nefropatia membranosa, nefroesclerose maligna e glomerulonefrite crônica entre os pacientes com dados disponíveis. Notavelmente, não foi relatada a presença de comorbidades significativas, imunossupressão ou outros problemas de saúde sistêmicos em nenhum dos pacientes, além da condição nefrológica subjacente que tornou necessária a DP. Isso pode sugerir que a peritonite associada à *Moraxella* também pode ocorrer em indivíduos imunocompetentes. Foi observado um histórico de episódios anteriores de peritonite na maioria dos casos (56%; 5/9), envolvendo tipicamente patógenos comuns, como *Staphylococcus aureus* ou espécies de *Streptococcus*.

**Tabela 1:** Casos relatados de infecções associadas à diálise peritoneal causadas por espécies de *Moraxella*, listados cronologicamente do mais antigo ao mais recente

No.	Ano de Publicação, Idioma, Referência	País	Idade (a)	Sexo	Tipo de infecção	Tempo em DP (meses), (etiologia)	Método de DP	Peritonite prévia
1	1987, Inglês^10^	UK	51	F	Peritonite	60 (nefropatia diabética)	CAPD	NA
2	1990, Inglês^11^	EUA	22	F	Peritonite	NA	CAPD	2 episódios de peritonite *(Staphylococcus aureus)*
3	1990, Inglês^11^	EUA	65	F	Peritonite	NA	CAPD	1 episódio de peritonite *(Staphylococcus aureus)*
4	1993, Inglês^12^	EUA	46	M	Peritonite	48 (nefropatia diabética)	CAPD	2 episódios de peritonite (1^o^ *Staphylococcus haemolyticus,* 2^o^ estreptococo a-hemolítico)
5	2003, Inglês^13^	EUA	48	M	Peritonite	NA	CAPD	NA
6	2009, Inglês^14^	Itália	70	F	Peritonite	60 (doença renal hipertensiva)	CCPD	3 episódios de peritonite bacteriana
7	2012, Inglês^6^	EUA	56	M	Peritonite	NA	DP	Não
8	2014, Inglês^1^	Países Baixos	47	M	Peritonite	53 (nefropatia membranosa)	CAPD	1 episódio de peritonite *(Streptococcus gordonii,* 36 meses antes)
9	2014, Inglês^15^	Espanha	73	M	Peritonite	24 (nefropatia diabética)	DP	NA
10	2016, Spanish^16^	Espanha	83	M	Peritonite	NA	DP	NA
11	2018, Inglês^17^	EUA	68	M	Peritonite	24 (NA)	D PA	Não
12	2018, Inglês^18^	Sérvia	59	M	Peritonite	12 (nefropatia diabética)	CAPD	Não
13	2019, Inglês^2^	Japão	26	M	Peritonite	14 (nefroesclerose maligna)	CAPD	Não
14	2024, English^3^	Japão	46	F	Peritonite	84 (glomerulonefrite crônica)	DP	NA

Abreviaturas - DP: diálise peritoneal; F: feminino; M: masculino; NA: não disponível; CAPD: diálise peritoneal ambulatorial contínua; CCPD: diálise peritoneal cíclica contínua; DPA: diálise peritoneal ambulatorial.

Entre os 14 pacientes com peritonite associada à *Moraxella*, havia informações disponíveis sobre a presença de febre em 12 casos. Destes, 7 (58%) estavam afebris, enquanto 5 (42%) apresentavam respostas febris, com temperaturas variando de 37,8 a 38,6 °C ou simplesmente registradas como “febre”. As contagens de leucócitos (WBC) no líquido peritoneal apresentaram variabilidade considerável, com valores entre 260 e 10.528 células/mm^3^. Observouse uma predominância de células polimorfonucleares (PMNs) em 8 casos (>90%), com a maior proporção registrada chegando a 99%. Um quadro apresentou uma porcentagem notavelmente baixa de PMNs (15%), e as contagens diferenciais não estavam disponíveis em dois casos. As contagens de WBC no sangue periférico estavam disponíveis em apenas 6 pacientes e variaram de 1.675 a 10.500 × 10^6^/L. Na maioria desses casos, observou-se predominância neutrofílica, com porcentagens de PMNs entre 78% e 92%, exceto em um paciente com uma proporção de neutrófilos notavelmente baixa (37%).

*M. catarrhalis* foi o isolado predominante (n = 6), seguida por *M. osloensis* (n = 5), enquanto *Moraxella phenylpyruvica* e *Moraxella nonliquefaciens* foram identificadas em um único caso cada (Tabela 2). Um caso adicional envolveu uma espécie não identificada de *Moraxella*. Métodos convencionais de cultura apoiaram o crescimento bacteriano (por exemplo, sistemas BacT/Alert, ágar chocolate, ágar sangue), seguidos pela identificação da espécie através de perfis bioquímicos ou sistemas automatizados de identificação (n = 10). Em relatos mais recentes, técnicas confirmatórias avançadas, como MALDI-TOF MS (n = 4) e/ou sequenciamento do gene 16S rRNA (n = 3), foram adicionalmente empregadas, refletindo a evolução das capacidades de diagnóstico microbiológico ao longo do tempo. Cinco casos relataram infecções polimicrobianas, incluindo o coisolamento de *Enhydrobacter aerosaccus*, *Kluyvera ascorbata*, *Rhizobium radiobacter*, *Streptococci/Enterococci* mistos e difteroides. Esses casos enfatizaram a necessidade de exames microbiológicos abrangentes, particularmente em pacientes com quadros clínicos atípicos. Os dados de sensibilidade aos antibióticos estavam disponíveis para 11 isolados. A maioria dos isolados apresentou suscetibilidade às cefalosporinas de terceira geração (p. ex., cefotaxima, ceftriaxona, ceftazidima), aos aminoglicosídeos (p. ex., gentamicina, tobramicina, amicacina), aos macrolídeos (p. ex., eritromicina, claritromicina) e às fluoroquinolonas (p. ex., ciprofloxacina, levofloxacina). No entanto, observou-se resistência à penicilina e à ampicilina em alguns casos, particularmente entre as cepas de *M. catarrhalis* e *M. osloensis*, o que é consistente com a conhecida produção de enzimas β-lactamases por essas espécies.

**Tabela 2:** Análise dos dados microbiologicos de 14 casos de peritonite relacionada à diálise peritoneal atribuídos a espécies de *Moraxella*

No (ref)	Isolado de *Moraxella*	Início do tratamento (d)	Método de isolamento	Coinfecção	Padrão de sensibilidade antimicrobiana	Padrão de resistência antimicrobiana
1^10^	*Branhamella catarrhalls*	1	Cultura do efluente da DP	Não	penicilina, cefuroxima, gentamicina, eritromicina, cloranfenicol	NA
2^11^	*Branhamella catarrhalls*	3	Cultura do efluente da DP	Não	ampicilina, cefalotina, tetraciclina, amicacina, gentamicina, tobramicina, mezlocilina, piperacilina, cloranfenicol	trimetoprima/sulfametoxazol
3^11^	*Branhamella catarrhalls*	1	Cultura do efluente da DP	Não	cefotaxima, gentamicina, cloranfenicol, trimetoprima/sulfametoxazol	ampicilina, penicilina, vancomicina
4^12^	*Moraxella catarrhalls*	2	Cultura do efluente da DP (BacT/Alert, ágar chocolate, ágar sangue)	Não	amoxicilina/ácido clavulânico, ampicilina/sulbactam, cefotaxima, ceftriaxona, cefuroxima, imipenem, eritromicina, cloranfenicol, gentamicina, trimetoprima/sulfametoxazol	ampicilina
5^13^	*Moraxella unidentified specie*	NA	Cultura do efluente da DP	*Kluyvera ascorbata*	NA	NA
6^14^	*Moraxella phenylpyruvlca*	NA	Cultura do efluente da DP, Versa TREK, BBL Crystal ID	Não	NA	NA
7^6^	*Moraxella catarrhalls*	14	Cultura do efluente da DP (ágar chocolate, ágar sangue)	Difteroides	amoxicilina/ácido clavulânico, ceftazidima, ceftriaxona, cefuroxima, claritromicina, ciprofloxacina, levofloxacina, tetraciclina, trimetoprima/sulfametoxazol	NA
8^1^	*Moraxella osloensls*	7	MALDI-TOF MS	*Rhlzoblum radlobacter*	Todos os antimicrobianos	Não
9^15^	*Moraxella nonllquefaclens*	NA	Cultura do efluente da DP (BacT/Alert, ágar chocolate, ágar sangue), Vitek-2, sequenciamento do gene 16S rRNA	Não	amoxicilina/ácido clavulânico, cotrimoxazol, azitromicina	NA
10^16^	*Moraxella osloensls*	2	MALDI-TOF MS	*Streptococci/Enterococci*	amoxicilina, amoxicilina/ácido clavulânico, cefuroxima, cefotaxima, ceftazidima, ciprofloxacina, gentamicina, azitromicina	vancomicina, teicoplanina, daptomicina
1^17^	*Moraxella osloensls*	1	Sequenciamento do gene 16S rRNA	Não	NA	NA
12^18^	*Moraxella catarrhalls*	1	Cultura do efluente da DP (BacT/Alert)	Não	ampicilina, amoxicilina/ácido clavulânico, cefazolina, ceftazidima, ceftriaxona, eritromicina, trimetoprima/sulfametoxazol, tetraciclina	vancomicina
13^2^	*Moraxella osloensls*	2	MALDI-TOF MS, sequenciamento do gene 16S rRNA	*Enhydrobacter aerosaccus*	ampicilina, cefaclor, cefotiam, ceftriaxona, cefepima, imipenem, claritromicina, levofloxacina, minociclina	clindamicina
14^3^	*Moraxella osloensls*	10	MALDI-TOF MS	Não	ampicilina/sulbactam, cefotaxima, ceftazidima, ceftriaxona, cefepima, imipenem, meropenem, eritromicina, claritromicina, levofloxacina	penicilina G, ampicilina, clindamicina, vancomicina, linezolida, daptomicina

Abreviaturas - DP: diálise peritoneal; NA: não disponível; BacT/Alert e Versa TREK: sistemas automatizados de detecção microbiana; BBL Crystal ID e Vitek-2: sistemas de identificação microbiana; MALDI-TOF MS: espectrometría de massas por ionização/dessorção a laser assistida por matriz com tempo de voo; sequenciamento do gene 16S rRNA: sequenciamento do gene do RNA ribossômico 16S.

Em infecções polimicrobianas, a escolha da terapia antibiótica foi orientada pelo organismo coisolado com maior resistência. Notavelmente, em um caso envolvendo coisolamento com *Rhizobium radiobacter*, a resistência a múltiplos β-lactâmicos tornou necessário o uso de ciprofloxacina intraperitoneal e meropenem por via intravenosa.

Todos os pacientes com dados completos registrados receberam antibióticos por via intraperitoneal como parte do protocolo de tratamento inicial. Os agentes mais comumente utilizados (p. ex., cefazolina, ceftazidima) foram frequentemente administrados em combinação com aminoglicosídeos ou fluoroquinolonas (Tabela 3). A duração do tratamento variou de 10 a 28 dias, com mediana de 21 dias. A remoção do cateter foi necessária em apenas dois casos, um envolvendo *Moraxella phenylpyruvica* e o outro, *M. osloensis*, ambos resultando em uma transição para hemodiálise. Em todos os 14 casos, a infecção foi resolvida sem mortalidade ou complicações a longo prazo. É importante destacar que a preservação do cateter foi alcançada em 86% (12/14) dos pacientes, reafirmando o prognóstico geralmente favorável das infecções por *Moraxella* relacionadas à DP quando prontamente reconhecidas e adequadamente tratadas.

**Tabela 3:** Estratégias terapêuticas e desfechos clínicos nos casos revisados de peritonite associada a *Moraxella* em pacientes em diálise peritoneal

No.^(ref)^	Remoção do cateter	Transição para hemodiálise	Tratamento (duração)	Desfecho
1^10^	Não	Não	cefuroxima ip + gentamicina ip (10 dias)	Recuperado
2^11^	Não	Não	vancomicina ip + vancomicina iv ≥ penicilina G im + penicilina G ip	Recuperado
3^11^	Não	Não	vancomicina ip + vancomicina iv ≥ gentamicina iv + gentamicina ip + trimetoprima/sulfametoxazol ip + trimetoprima/sulfametoxazol iv + ceftriaxona iv	Recuperado
4^12^	Não	Não	cefazolina im + tobramicina im ≥ cefazolina ip (10 dias) + tobramicina ip	Recuperado
5^13^	Não	Não	levofloxacina + tobramicina ip (14 dias)	Recuperado
6^14^	Sim (dia 5)	Sim	tobramicina ip ≥ vancomicina + fluconazol ≥ cefixima pos	Recuperado
7^6^	Não	Não	vancomicina ip + ciprofloxacina iv ≥ vancomicina ip + ceftazidima ip (21 dias)	Recuperado
8^1^	Sim (dia 12)	Sim	cefazolina ip ≥ ceftazidima ip ≥ ciprofloxacina ip ≥ meropenem iv	Recuperado
9^15^	Não	Não	cefazolina ip + ceftazidima ip ≥ ceftazidima ip (28 dias)	Recuperado
10^16^	Não	Não	cefazolina ip + ceftazidima ip + linezolida pos ≥ ceftazidima ip + amoxicilina/ácido clavulânico pos	Recuperado
11^17^	Não	Não	ceftazidima ip (21 dias)	Recuperado
12^18^	Não	Não	cefazolina ip + amicacina ip ≥ cefazolina ip (14 dias)	Recuperado
13^2^	Não	Não	ceftazidima ip + cefazolina ip ≥ cefazolina ip (21 dias)	Recuperado
14^3^	Não	Não	ceftazidima ip + cefazolina ip ≥ ceftazidima ip (21 dias)	Recuperado

Abreviaturas – ip: intraperitoneal; iv: intravenoso; im: intramuscular; pos: *per os*.

O prognóstico global foi favorável em todos os casos relatados. Não foi documentada qualquer mortalidade relacionada à infecção. A resolução clínica dos sintomas de peritonite foi geralmente alcançada poucos dias após o início da antibioticoterapia. Os parâmetros laboratoriais, incluindo a contagem de leucócitos no líquido peritoneal e os marcadores inflamatórios séricos, demonstraram uma rápida melhora na maioria dos casos.

## Discussão

A presente revisão sistemática examina de forma abrangente o papel das espécies de *Moraxella* como agentes etiológicos em infecções associadas à diálise peritoneal, com foco específico na peritonite. A documentação exclusiva de casos relacionados à *Moraxella* como peritonite enfatiza sua baixa frequência clínica, as complexidades diagnósticas inerentes e os desfechos terapêuticos geralmente favoráveis. Os achados destacam a importância de manter um alto índice de suspeita para patógenos atípicos, como as espécies de *Moraxella*, que, apesar de sua raridade, merecem reconhecimento clínico no diagnóstico diferencial da peritonite relacionada à DP. Sua identificação tem sido historicamente negligenciada, provavelmente devido às limitações das metodologias diagnósticas tradicionais e à sua natureza comensal em sítios mucosos.

Uma observação consistente em todos os casos foi a predominância de *M. catarrhalis* e *M. osloensis*, ambas tradicionalmente associadas à colonização do trato respiratório. O isolamento dessas espécies do líquido peritoneal levanta questões intrigantes acerca dos mecanismos patogênicos. Foram propostas diversas vias de colonização da cavidade peritoneal, incluindo contaminação direta durante as trocas de DP, translocação a partir do trato gastrointestinal e disseminação hematogênica a partir de focos mucosos distantes^3,6,7^. A presença de organismos coinfetantes em alguns casos pode sugerir uma origem polimicrobiana ou refletir comprometimento das barreiras mucosas.

É importante ressaltar que a revisão destaca a evolução do arsenal microbiológico na detecção de espécies de *Moraxella*. Enquanto os casos mais antigos baseavam-se exclusivamente em testes bioquímicos e no crescimento em meios seletivos, os casos mais recentes incorporaram a MALDI-TOF MS e o sequenciamento do gene 16S rRNA para identificação precisa em nível de espécie^19^. Esses avanços aumentaram significativamente a sensibilidade e a especificidade da detecção de patógenos e podem explicar, em parte, o aumento da taxa de detecção de espécies de *Moraxella* nos últimos anos^2^. No entanto, apesar do progresso tecnológico, atrasos no diagnóstico podem ocorrer em ambientes com recursos limitados. Do ponto de vista clínico, é necessária uma maior conscientização para o reconhecimento precoce da peritonite por *Moraxella* associada à DP. Infecções causadas por organismos fastidiosos podem apresentar sintomas subagudos ou perfis laboratoriais atípicos, levando a classificações incorretas ou subdiagnósticos.

O perfil de suscetibilidade antimicrobiana dos isolados de *Moraxella* em infecções associadas à DP foi, em geral, favorável. A maioria das cepas apresentou suscetibilidade às cefalosporinas, aos aminoglicosídeos e às fluoroquinolonas, em consonância com os perfis antibióticos já conhecidos das espécies de *Moraxella*. Em particular, a *M. catarrhalis*, apesar de sua capacidade de produzir enzimas β-lactamase, demonstrou suscetibilidade preservada às cefalosporinas. No entanto, foi relatada resistência à penicilina e à ampicilina em alguns isolados tanto de *M. catarrhalis* quanto de *M. osloensis*, ressaltando a importância da terapia antimicrobiana individualizada orientada por testes de suscetibilidade^2,5^.

Apesar da presença de isolados com suscetibilidade variável aos antibióticos, os desfechos clínicos foram uniformemente favoráveis, com resolução da infecção em todos os 14 pacientes. A maioria das infecções foi manejada com sucesso sem a necessidade de remoção do cateter, um desfecho significativo considerando que a preservação do cateter é um determinante crítico para a continuidade da DP a longo prazo. Somente dois pacientes (14%) necessitaram da remoção do cateter, e ambos acabaram retomando a diálise peritoneal após a transição para a hemodiálise. Esse dado reafirma observações prévias de que mesmo patógenos não tradicionais podem ser efetivamente manejados com intervenção precoce e terapia adequada^20^.

As estratégias terapêuticas variaram quanto à escolha do medicamento e à duração do tratamento, refletindo decisões empíricas adaptadas à condição do paciente e às práticas locais. Notavelmente, as cefalosporinas por via intraperitoneal, frequentemente em combinação com aminoglicosídeos ou fluoroquinolonas, constituíram os regimes mais comumente empregados. A duração do tratamento esteve tipicamente alinhada às diretrizes padrão da ISPD para peritonite por bactérias gram-negativas, variando de 14 a 21 dias^8^. A recuperação consistente observada em nossa coorte sugere que as infecções por *Moraxella* em pacientes em DP seguem um curso benigno quando manejadas de acordo com o protocolo.

Um ponto adicional a ser considerado é o potencial impacto das infecções polimicrobianas. Em 5 dos 14 casos analisados (~36%), espécies de *Moraxella* foram coisoladas com outros microrganismos em culturas de líquido peritoneal, dificultando a interpretação dos achados microbiológicos e a determinação do agente causador primário. Os organismos coisolados, incluindo *Enhydrobacter aerosaccus*, *Kluyvera ascorbate* e *Rhizobium radiobacter*, são raramente relatados ou pouco caracterizados como agentes etiológicos na peritonite relacionada à DP. Entre eles, a *R. radiobacter* tem sido ocasionalmente implicada nesse contexto, principalmente em hospedeiros imunocomprometidos ou no contexto de infecções relacionadas ao cateter^21^. Em contrapartida, a *K. ascorbata* e a *E. aerosaccus* são consideradas organismos de baixa virulência, com associações apenas anedóticas ou excepcionais com doenças humanas e praticamente nenhuma ligação estabelecida com infecções associadas à DP. *Streptococci/Enterococci* mistos e os difteroides, embora sejam mais frequentemente encontrados em amostras clínicas, são muitas vezes considerados contaminantes, apesar de apresentarem potencial patogenicidade em casos selecionados^8^. Como resultado, o papel patogênico da *Moraxella* nesses episódios polimicrobianos permanece incerto. Sem dados clínicos, inflamatórios e microbiológicos mais detalhados, não é possível determinar se ela atua como um patógeno verdadeiro ou apenas como um comensal. No entanto, essas infecções mistas podem obscurecer o diagnóstico e retardar a instituição de terapia direcionada. É importante ressaltar que, em todos os casos polimicrobianos, o tratamento antimicrobiano orientado por cultura levou à resolução clínica, ressaltando a importância crítica de uma avaliação microbiológica minuciosa, particularmente em casos com apresentações atípicas ou resposta inicial insatisfatória à terapia empírica^22^.

Nosso estudo apresenta certas limitações inevitáveis, principalmente devido ao fato de se basear em relatos de casos previamente publicados, em vez de dados originais de pacientes. Como resultado, está sujeito a viés de publicação e pode não representar a gama completa de cenários clínicos. Além disso, a heterogeneidade nos relatos, particularmente no que se refere aos testes de suscetibilidade e aos esquemas de tratamento, pode limitar a generalização. No entanto, a presente revisão fornece a análise mais abrangente até o momento sobre a peritonite por *Moraxella* na DP e oferece orientações clinicamente relevantes.

Em conclusão, embora raras, as espécies de *Moraxella* são patógenos reconhecidos no contexto da peritonite associada à DP. O diagnóstico deve basear-se nas características clínicas, na análise do líquido peritoneal e nos resultados da cultura. A identificação imediata utilizando métodos de cultura convencionais, com técnicas confirmatórias avançadas quando disponíveis, juntamente com terapia antimicrobiana adequada e manejo criterioso do cateter, pode resultar em desfechos clínicos uniformemente favoráveis. À medida que as ferramentas diagnósticas se tornam mais sofisticadas, os médicos devem considerá-las e integrá-las à prática laboratorial. São necessárias pesquisas adicionais para definir a real incidência de infecções por *Moraxella* relacionadas à diálise peritoneal, as estratégias de manejo ideais e o impacto a longo prazo nos desfechos da diálise peritoneal.

## Data Availability

Nenhum dado novo foi gerado ou analisado neste estudo.
